# Probing Origin of Binding Difference of inhibitors to MDM2 and MDMX by Polarizable Molecular Dynamics Simulation and QM/MM-GBSA Calculation

**DOI:** 10.1038/srep17421

**Published:** 2015-11-30

**Authors:** Jianzhong Chen, Jinan Wang, Qinggang Zhang, Kaixian Chen, Weiliang Zhu

**Affiliations:** 1 School of Science, Shandong Jiaotong University, Jinan, 250014, China; 2 Discovery and Design Center, CAS Key Laboratory of Receptor Research, Shanghai Institute of Materia Medica, Chinese Academy of Sciences, 555 Zuchongzhi Road, Shanghai, 201203, China; 3 College of Physics and Electronics, Shandong Normal University, Jinan, 250014, China

## Abstract

Binding abilities of current inhibitors to MDMX are weaker than to MDM2. Polarizable molecular dynamics simulations (MD) followed by Quantum mechanics/molecular mechanics generalized Born surface area (QM//MM-GBSA) calculations were performed to investigate the binding difference of inhibitors to MDM2 and MDMX. The predicted binding free energies not only agree well with the experimental results, but also show that the decrease in van der Walls interactions of inhibitors with MDMX relative to MDM2 is a main factor of weaker bindings of inhibitors to MDMX. The analyses of dihedral angles based on MD trajectories suggest that the closed conformation formed by the residues M53 and Y99 in MDMX leads to a potential steric clash with inhibitors and prevents inhibitors from arriving in the deep of MDMX binding cleft, which reduces the van der Waals contacts of inhibitors with M53, V92, P95 and L98. The calculated results using the residue-based free energy decomposition method further prove that the interaction strength of inhibitors with M53, V92, P95 and L98 from MDMX are obviously reduced compared to MDM2. We expect that this study can provide significant theoretical guidance for designs of potent dual inhibitors to block the p53-MDM2/MDMX interactions.

The tumor suppressor protein p53 plays a key role in maintaining genetic integrity and preventing tumor development^1^. Upon cellular stress, p53 can be stabilized and activate pathways that either mediate cell-cycle arrest, senescence or apoptosis^2^. Loss of p53 function caused by mutations is involved in 50% of cancer patients in the world. In the remainder, although p53 retains wild-type function, its signaling pathway is inactivated via the interactions with two oncoproteins MDM2 and MDMX^3^,^4^,^5^,^6^. The previous studies have proved that the restoration of p53 activity can efficiently inhibit the growth of cancerous tumors in animals^7^,^8^,^9^. Thus, designs of chemical compounds, activating the p53 signaling pathway by inhibition of the p53-MDM2/MDMX interaction, is an important channel of cancer therapy.

Recent studies showed that sequence identity in the N-terminal domain of MDM2 and MDMX reaches ~54%, and these two oncoproteins share highly similar overall structure (Fig. 1A)^10^,^11^,^12^. Both MDM2 and MDMX can produce direct interactions with the residues F19′, W23′ and L26′, located in the TA domain of p53, which contribute a majority of binding free energies of p53 to MDM2/MDMX^4^,^13^,^14^,^15^,^16^. Up to now, many chemical compounds, such as peptide inhibitors PMI^17^, P4^18^, pDI6W^19^ etc. and non-peptide inhibitors nutlins^20^, isoindolinone^21^, spiro-oxindoles (MI-63)^22^,^23^ and benzodiazepinedions derivatives^24^, have been developed by mimicking the p53-MDM2 interaction. Although these compounds have high binding affinities to MDM2, they cannot efficiently inhibit the p53-MDMX interaction. Moreover, inhibitor bindings can induce large conformational changes of MDM2 and MDMX^25^,^26^, especially for MDMX, which plays a significant role in clarification of the structure-affinity relationship for the inhibitor-MDM2/MDMX complex. Thus, it is significant to probe origin of the differences in binding modes and conformational changes induced by inhibitor bindings at an atomic level for designs of potent dual inhibitors targeting the p53-MDM2/MDMX interactions.

Due to great success of molecular simulations and predictions of binding free energies in insight into the inhibitor-protein interactions, conformation changes and structure-affinity relationship of proteins^27^,^28^,^29^,^30^,^31^,^32^,^33^,^34^,^35^,^36^,^37^,^38^,^39^,^40^,^41^,^42^,^43^,^44^,^45^, these methods were also applied to study interaction mechanism of inhibitors with MDM2 and MDMX. For example, quantum mechanics method was adopted by Ding *et al*. to calculate residue-specific interactions in the p53-MDM2 complex and the results proved that van der Waals interactions drive the p53-MDM2 binding^46^, which was also supported by previous calculated studies of other groups^47^,^48^,^49^. Joseph *et al*. used MD simulations and binding free energy calculations to study the binding modes of p53 and nutlin to MDM2/MDMX and their results suggested that p53 and nutlin have higher affinity for MDM2 than MDMX^50^. Alanine scanning mutagenesis calculations performed by several groups indicated that four key position mutations (Phe19, Leu22, Trp23 and Leu26) in p53 led to a significant decrease in binding free energies^51^,^52^,^53^. Espinoza-Fonseca *et al*. combined MD simulation with principal component (PC) analysis to explore the conformation changes of MDM2 and their results showed that the binding cleft of MDM2 undergoes a large conformational change^25^.

Compared to classical molecular force field, polarizable force field plays more important role in stabilization of protein structure^54^,^55^,^56^,^57^,^58^. The polarized protein-specific charge (PPC) developed by Zhang’s group not only stabilized intra-protein hydrogen bonding interactions, but also was used to accurately predict binding free energy^59^,^60^,^61^. Jiao *et al*. successfully integrated AMOEBA polarizable force field with binding free energy calculation to investigate binding modes of inhibitors to trypsin^62^. Patel *et al*. applied MD simulation involving CHARMM fluctuating charge force field to investigate the structures of six small proteins and the results suggested that these proteins maintained native structures during simulation^63^. Thus, polarizable MD simulation will be helpful to clarify the difference in the binding mode of inhibitors to MDM2 and MDMX.

In this work, two peptide inhibitors pDI6W (LTFEHWWAQLTS) and pDIQ (ETFEHWWSQLLS) were selected to probe interaction mechanism of inhibitors with MDM2 and MDMX at the atomic level. The median inhibitory concentration (IC50) values of pDI6W to MDM2 and MDMX are 36 and 250 nM, respectively, and the corresponding values of pDIQ to MDM2 and MDMX are 8 and 110 nM^19^, which shows that binding ability of two inhibitors to MDM2 is obviously stronger than MDMX. Based on success of Amber FF02 polarizable force field in insight into other protein systems^64^,^65^,^66^, polarizable (FF02) and non-polarizable (FF03) MD simulations were carried out to investigate the conformation changes of MDM2/MDMX. In addition, QM/MM-GBSA^67^,^68^,^69^,^70^ and solvated interaction energy (SIE)^71^ methods were adopted to calculate binding free energies of pDI6W and pDIQ to MDM2/MDMX and reveal the origin of binding difference of inhibitors to MDM2 and MDMX. We expect that this study can contribute an important theoretical guidance and dynamic information for development of potent dual inhibitors blocking the p53-MDM2/MDMX interactions.

## Results

### Polarizable MD simulation vs non-polarizable MD simulations

To evaluate the effect of polarizable and non-polarizable MD simulation on the stability of structure, the RMSD values of all backbone atoms in the complexes were calculated relative to their corresponding crystal structures and plotted in Figure S1 in the supporting information. Figure S1 shows that all of four polarizable MD simulations reach the equilibrium at ~10 ns of simulations, while non-polarizable MD simulations do not tend to the equilibrium until 15 ns of simulations. After the equilibrium, the average RMSD values of four polarizable MD simulation are lower than 1.06 Å, but the corresponding RMSD values of four non-polarizable MD simulation are higher than 1.38 Å. Moreover the fluctuating range of four polarizable MD simulations after the equilibrium are smaller than that of four non-polarizable MD simulations.

The combination of the Cpptraj program^72^ and the DSSP second structure analysis^73^ was applied to estimate the stability of the second structure of MDM2 and MDMX in two different-type simulations. Figure 2 and Figure S2 describe the time evolution of the secondary structure profiles of MDMX and MDM2 in the polarizable and non-polarizable MD simulations. One can observe that the polarizable ff02.r1/POL3 combination can lead to a slightly more stable second structure relative to the non-polarizable ff03/TIP3P combination. This result agrees well with the earlier reports^65^,^74^. The time evolution of the number of hydrogen bonds in the complex through simulation is analyzed by using the Cpptraj program and plotted in Figure S3. The results show that the average number of hydrogen bonds in polarizable MD simulations are ~20 more than that in the non-polarizable MD simulations. This result gives a rational reason why the polarizable MD simulations can better stabilize the second structure. Based on the above results, all post-processing analyses are performed on the trajectories of polarizable MD simulations.

### Calculations of binding free energies

To measure the strength of interactions between inhibitors and MDM2/MDMX, the absolute binding free energies were calculated by using QM/MM-GBSA method implemented in Amber program^75^. In the inhibitor-MDM2/MDMX binding complexes, the residues involving the hydrogen bonds were treated using the semi-empirical Hamiltonian method PM6, the rest of complexes were calculated by molecular mechanics. The calculated results were listed in Table 1.

According to Table 1, the binding free energies (ΔG_bind_) of the pDI6W-MDMX, pDI6W-MDM2, pDIQ-MDMX, pDIQ-MDM2 complexes are −8.94, −11.02, −9.18 and −11.82 kcal · mol^−1^, respectively, which agrees well with the experimentally determined values. This result shows that the binding abilities of two inhibitors to MDMX are weaker than to MDM2. Additionally, the entropy changes (−TΔS) induced by inhibitor bindings produce a good correlation with the interaction enthalpy (ΔH_tot_). This result should be rational because the stronger the interaction enthalpy is, the more the restrictions of motion freedoms are.

As seen from Table 1, the van der Waals interaction (ΔE_vdW_), non-polar solvation energy (ΔG_surf_) and quantum mechanics energy (ΔG_qm_) provide favorable contribution to the inhibitor bindings. Although the electrostatic interaction (ΔE_ele_) also favors in the inhibitor bindings, this favorable factor are completely screened by the stronger unfavorable polar solvation energy (ΔG_gb_). Compared to the pDI6W-MDM2 complex, the van der Waals interaction, non-polar solvation energy and quantum mechanics energy of the pDI6W-MDMX binding are decreased by 2.5, 0.42 and 0.97 kcal · mol^−1^, respectively. Similarly, the pDIQ-MDMX binding also leads to the decrease of 2.65, 0.83 and 0.44 kcal · mol^−1^ in the van der Waals interaction, non-polar solvation energy and quantum mechanics energy, respectively. Overall, the decrease in the van der Waals should be a main origin of weaker binding abilities of inhibitors to MDMX than to MDM2.

To further support the above conclusion, the binding free energies were also computed by using the SIE method and the results were given in Table 2. One can see that the rank of predicted binding free energies agrees with the experimentally determined one. According to Table 2, the van der Waals interactions (ΔE_vdW_) and molecular surface-correlated energies (γ·ΔMSA) is favorable for the inhibitor bindings. Despite favorable contributions of the intermolecular Coulomb interactions (ΔE_c_), this favorable force was completely counteracted by the unfavorable reaction energies (ΔG^R^). Compared to the MDM2 binding complexes, the pDI6W-MDMX binding leads to the decrease of 3.72 and 0.45 kcal · mol^−1^ in ΔE_vdW_ and γ·ΔMSA, respectively, and for the pDIQ-MDMX binding, ΔE_vdW_ and γ·ΔMSA are reduced by 5.7 and 0.60 kcal · mol^−1^, respectively. Thus, the decrease in the van der Waals interactions is the main origin of less efficiency of inhibitors on MDMX than on MDM2. This result is in good agreement with the previous QM/MM-GBSA calculations. Studies of Joseph *et al*. proved that p53 and nutlin also produce weaker affinity to MDMX than to MDM2^50^.

### Analysis of the structure-affinity relationship

To further explore the origin of weaker binding of inhibitors to MDMX than to MDM2, the inhibitor-residue interaction were calculated by using the residue-based free energy decomposition method and the results depicted in Fig. 3. The relative positions of the residues involving important contributions in the complexes were described in Fig. 4 by using the lowest energy structure from the polarizable MD trajectory. The information of hydrogen bonds from the Cpptraj analysis was listed in Table 3.

According to Fig. 3A, the interactions of 11 residues in MDM2 with the inhibitor pDI6W are stronger than −1.30 kcal · mol^−1^. These residues involve K51, L54, I61, M62, Y67, Q72, H53, V93, H96, I99 and Y100. One can observe that the residue L54 produce the strongest interaction (−4.27 kcal · mol^−1^) with pDI6W. This interaction mainly arises from two contributions, one from the CH-CH interactions between the alkyls of L54 and L26′, another from the hydrogen bonding interaction between the atom NE1 of W23′ and the carbonyl oxygen O of L54 (Fig. 4A), with a distance of 2.87 Å and the occupancy of 99.09% (Table 3). Figure 4A shows that the alkyls of I61 and M62 are located near the phenyl of F19′ and easy to form the CH-π interactions^76^, which provide contributions of −1.69 and −2.06 kcal · mol^−1^ to the pDI6W binding (Fig. 3A), respectively. The interactions of Y67 and H73 with pDI6W are −2.16 and −2.12 kcal · mol^−1^, respectively, which structurally agree well with the CH-π interactions of the phenol in Y67 and the imidazole in H73 with the phenyl of F19′. For the residue Q72, not only its CH group can generate the CH-π interaction with F19′, but also its oxygen OE1 can form a hydrogen bonding interaction with the N-H of backbone in F19′ (Table 3), which provide a total contribution of −1.42 kcal · mol^−1^ to the inhibitor binding. The interactions of V93 and I99 with pDI6W are −3.19 and −1.94 kcal · mol^−1^, respectively (Fig. 3A). The pDI6W-V93 interaction mostly comes from the CH-π interaction between the alkyls of V93 and the indole of W23′, while the pDI6W-I99 interaction is contributed by the CH-CH interactions between the alkyls of V93 and L26′ (Fig. 4A). Structurally, the imidazole of H96 and the phenol of Y100 are close to the alkyls of L26′ to form the CH-π interactions, which contribute interaction energies of −2.22 and −1.37 kcal · mol^−1^ to the pDI6W binding, respectively. The interaction of K50 with PDI6W is −2.89 kcal · mol^−1^, and this interaction mainly arises from the CH-O interactions between the CH groups of K51 and the carbonyl O of T27′.

By comparison with the pDI6W-MDM2 complex, the binding mode of pDI6W to MDMX is basically similar to that of pDI6W to MDM2 (Fig. 3B). However, the inhibitor-residue interaction strength changes obviously. The interactions of pDI6W with K50 and Q71 of MDMX are strengthened by 1.1 and 1.3 kcal · mol^−1^ than that of pDI6W with K51 and Q72 of MDM2, respectively. The binding efficiency of pDI6W to M53 and Y66 in MDMX were decreased by 1.58 and 0.63 kcal · mol^−1^ relative to that of pDI6W with L54 and Y67 in MDM2. As shown in Fig. 3A,B, although the pDI6W-Y99 interaction is increased by 0.59 kcal · mol^−1^ compared to Y100 in MDM2, the interaction intensity of pDI6W with V92, P95 and L98 of MDMX are reduced by 0.56, 1.29 and 0.96 kcal · mol^−1^ relative to the one of pDI6W with V93, H96 and I99 of MDM2, respectively. In addition, the occupancy of the hydrogen bonds between pDI6W and the residues M53 and Q71 were also decreased relative to that between pDI6W and the residues L54 and Q72 (Table 3), which is supported by the decrease in the peak values of the radial distribution function of H–O distance for hydrogen bonds (Figure S4A and B).

Figure 3C,D suggest that the binding modes of pDIQ to MDM2 and MDMX are similar to that of pDI6W to MDM2 and MDMX. Thus, the analyses of the interaction details of pDIQ with MDM2/MDMX are also similar to pDI6W. By comparing Fig. 3C with 3D, the binding difference of pDIQ to MDM2 and MDMX can be identified. Except for the obvious increase of the interactions of pDIQ with M61 and Y99 in MDMX relative to that of pDIQ with M62 and Y100 in MDM2, the interaction strengths of pDIQ with K50, M53, Y66, V92, P95 and L98 in MDMX are reduced by 0.81, 0.82, 0.31, 1.22, 0.48 and 1.11 kcal · mol^−1^ compared to that of pDIQ with K51, L54, Y67, V93, H96 and I99 in MDM2, respectively. Additionally, the occupancy of the hydrogen bonds between pDIQ and the residues M53 and Q71 in MDMX are lower than that between pDIQ and the residues L54 and Q72 (Table 3), which also agrees well with the decrease in the peak values of the radial distribution function of H–O distance for hydrogen bonds (Figure S4C and D).

Based on the above analyses, one can observe that the interactions of inhibitors with five common residues M53, Y66, V92, P95 and L98 in MDMX are obviously weaker than the ones of inhibitors with the corresponding residues in MDM2, which provides a main contributions to the weaker binding of inhibitors to MDMX than to MDM2. In addition, the hydrogen bonding interactions of inhibitors with the residues of MDMX are slightly weakened relative to MDM2, which rationally explains the decrease of quantum mechanics energy in the calculation of QM/MM-GBSA.

### Internal dynamics analyses

To quantitatively measure the mean backbone flexibility of separate residues, the RMSF values of C_α_ atoms in MDM2/MDMX were calculated using the equilibrium trajectory, as shown in Fig. 5. The results suggest that the mobility of the loop L3 (the residues 76–79), L5 (the residues 93–96) and the helix α4 domain (the residues 97–108) in MDMX is stronger than that in MDM2. Additionally, the domains near the residues M53 and Y66 in MDMX also produce greater flexibility relative to the corresponding domains in MDM2. The residues involving the decrease of the inhibitor-residue interactions in MDMX relative to MDM2 are located in the above domains, which shows that the changes in the inhibitor-residue interactions can significantly affect the mobility of proteins.

The inhibitor-Y99 interaction in MDMX is stronger than the inhibitor-Y100 interaction in MDM2. To reveal the reason of this change, the time evolution of the Chi1 (χ1) dihedral angle of the sidechain in Y99 (MDMX) and Y100 (MDM2) and their frequency distribution were calculated through the polarizable MD simulation (shown in Fig. 6). One can observe that the mobility of the side chain of Y99 is much stronger than that of Y100 (Fig. 6A,C). According to Fig. 6B,D, the frequency distribution of Chi1 dihedral angle of Y99 is located near 286°, while that of Y100 around 186°. This result shows that the orientation of the side chain for Y99 in MDMX is highly different from that of the side chain for Y100 in MDM2, which may significantly affect the inhibitor bindings.

The free energy landscapes were constructed by using the backbone ψ and φ angle to further probe the conformational change of Y99 (MDMX) relative to Y100 (MDM2), as shown in Fig. 7. The results show that the ψ and φ angle of MDMX generate changes relative to that of Y100. The ψ and φ angle of Y100 in the pDI6W-MDM2 complex are −47° and −58°, which are changed into −32° and −64° in the pDI6W-MDMX complex, respectively. Figure 7C,D suggest that the ψ and φ angle of Y100 correspond to −46° and −58° in the pDIQ-MDM2 complex, while for the pDIQ-MDMX complex, these two angles are transformed into −36° and −63°, respectively. One can note that the alternation of the ψ angle of Y99 in MDMX reaches 10° relative to that of Y100 in MDM2. For the rigid peptide bond, this alternations are great and must produce significant impact on the conformations of the residues nearby.

The residue L54 in MDM2 is substituted by M53 in MDMX and the length of the side chain of M53 is longer than the one of L54. To reveal how this change affect the inhibitor bindings, the time evolution of the Chi1 (χ1) dihedral angle of the side chain in M53 and L54 were calculated through the MD trajectory, as well as their frequency distribution were depicted in Fig. 8. The Chi1 (χ1) dihedral angle of M53 are much different from that of L54 during MD simulation. The Chi1 angle of L54 fluctuates around 285°, while the one of M53 frequently changes between two different states. The two peak values of the frequency distributions of M53 in the MDMX binding complexes are located in 195.4° and 286.2° respectively, while the single peak values of L54 in the MDM2 binding complexes are in 285.3°. In the MDMX binding complex, the population of the Chi1 for 195.4° is more than that of 286.2°. This result shows that the orientation of the side chain of M53 highly differs from the one of L54, which may produce significant effect on the inhibitor bindings. The free energy landscapes were constructed by using the backbone ψ and φ angle of M53 and L54 and plotted in Figure S5. The results indicate that the backbone ψ and φ angle of M53 hardly changes compared to that of L54.

**Figure 1:**
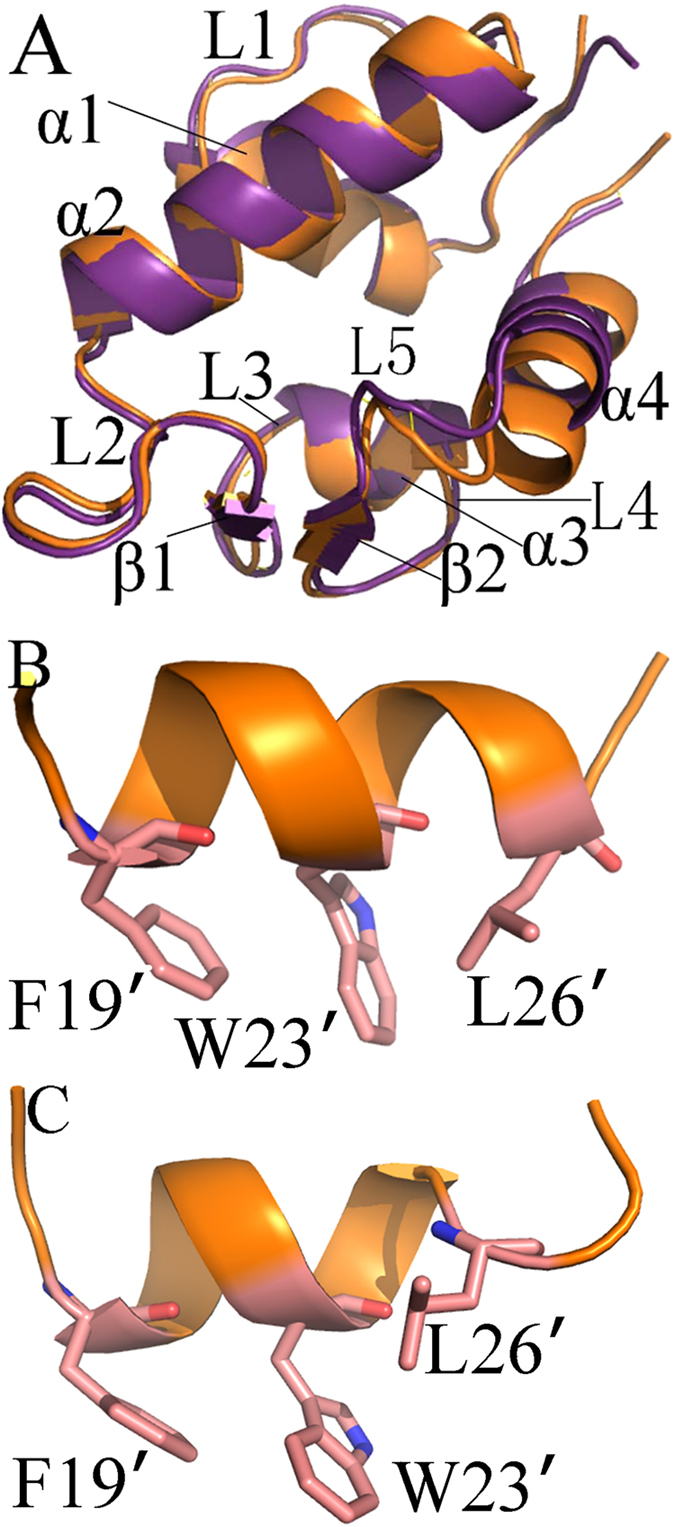
Structures of MDM2, MDMX and inhibitors: (**A**) superimposed structures of MDM2 and MDMX in a cartoon mode, MDM2 is shown in orange and MDMX in violetpurple; (**B**) structure for pDI6W and (**C**) structure for pDIQ.

**Figure 2:**
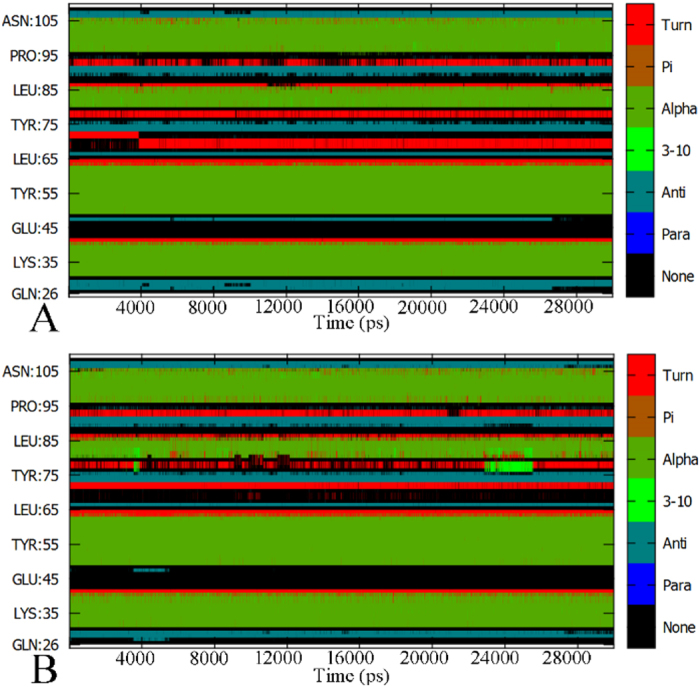
Time evolution of the secondary structure profile of MDMX in the pDI6W-MDMX complex: (**A**) for the polarizable ff02.r1/POL3 combination.(**B**) for the non-polarizable ff03/TIP3P combination; Anti and Para represent anti-parallel beta-sheet and parallel beta-sheet, respectively, while Pi, 3–10 and Alpha represent Pi (3–14) helix, 3–10 helix and alpha helix.

**Figure 3:**
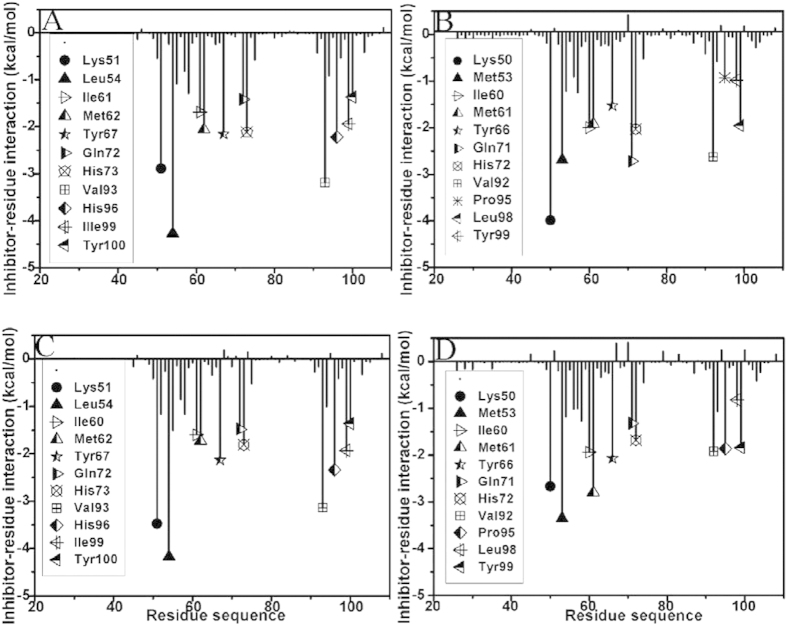
The inhibitor-residue interaction spectra: (**A**) the pDI6W-MDM2 complex, (**B**) the pDI6W-MDMX complex, (**C**) the pDIQ-MDM2 complex and (**D**) the pDIQ-MDMX complex.

**Figure 4:**
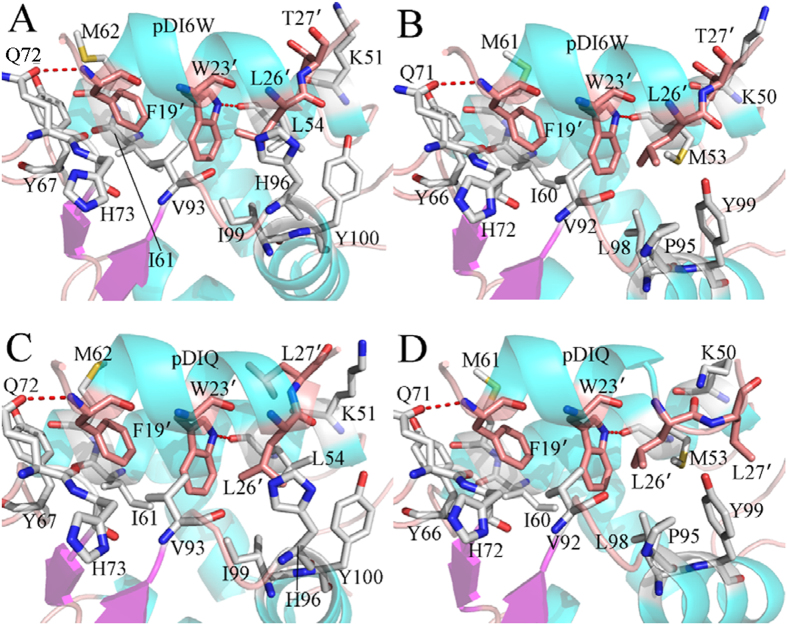
The relative position of the key residues in the binding complex: (**A**) the pDI6W-MDM2 complex, (**B**) the pDI6W-MDMX complex, (**C**) the pDIQ-MDM2 complex and (**D**) the pDIQ-MDMX complex.

**Figure 5:**
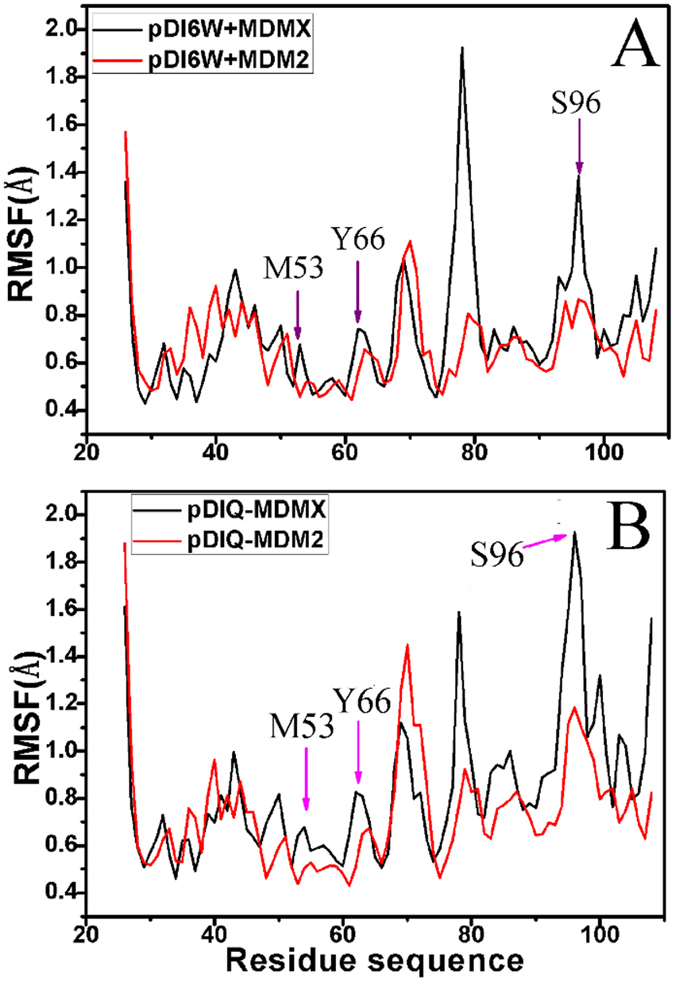
The RMSF of C_α_ atoms in MDMX and MDM2 through the equilibrium phase of MD simulation. (**A**) for the inhibitor pDI6W and (**B**) for the inhibitor pDIQ.

**Figure 6:**
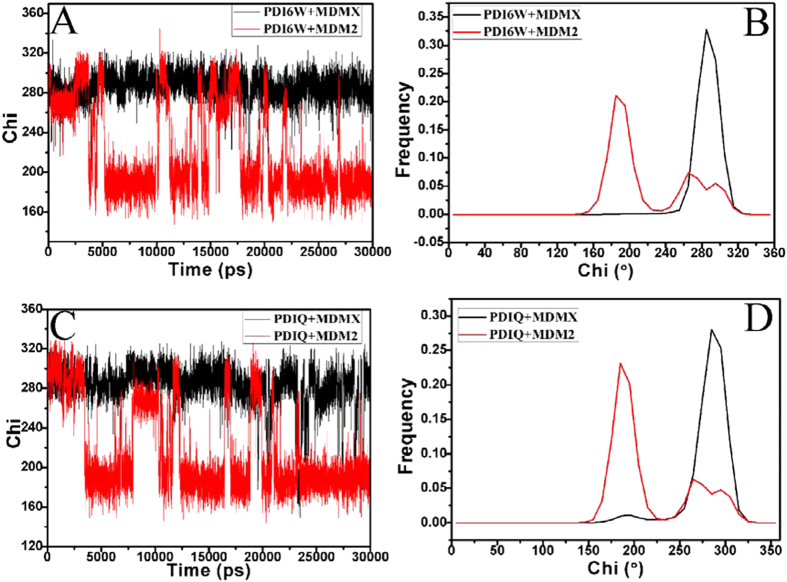
The Chi1 (χ1, in degree) dihedral angle of the side chain of Y100 in MDM2 and Y99 in MDMX as a function of time and frequency distribution: (**A,B**) for the pDI6W-MDM2/MDMX complexes, (**C,D**) for the pDIQ-MDM2/MDMX complexes.

**Figure 7:**
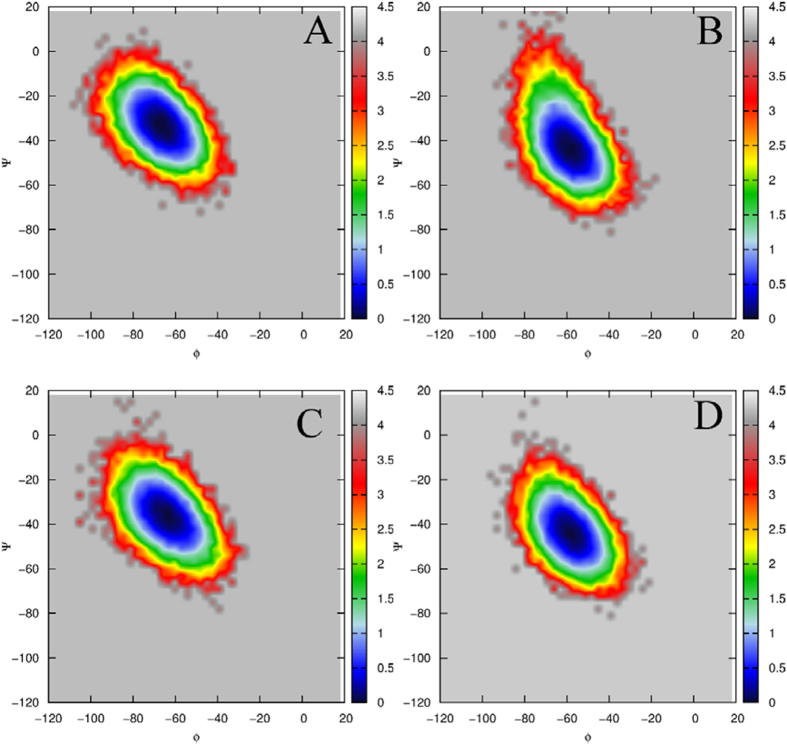
Free energy contour map as function of the backbone angle ψ and φ. (**A**) for Y100 in the pDI6W-MDM2 complex, (**B**) for Y99 in the pDI6W-MDMX complex. (**C**) for Y100 in the pDIQ-MDM2 complex and (**D**) for Y99 in the pDIQ-MDMX complex.

**Figure 8:**
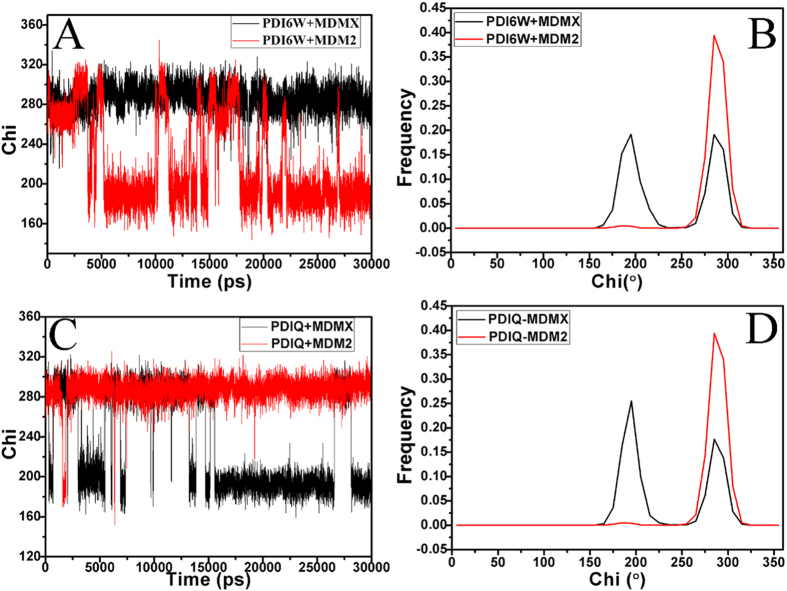
The Chi1 (χ1, in degree) dihedral angle of the side chain of L54 in MDM2 and M53 in MDMX as a function of time and frequency distribution: (**A,B**) for the pDI6W-MDM2/MDMX complexes, (**C,D**) for the pDIQ-MDM2/MDMX complexes.

**Figure 9:**
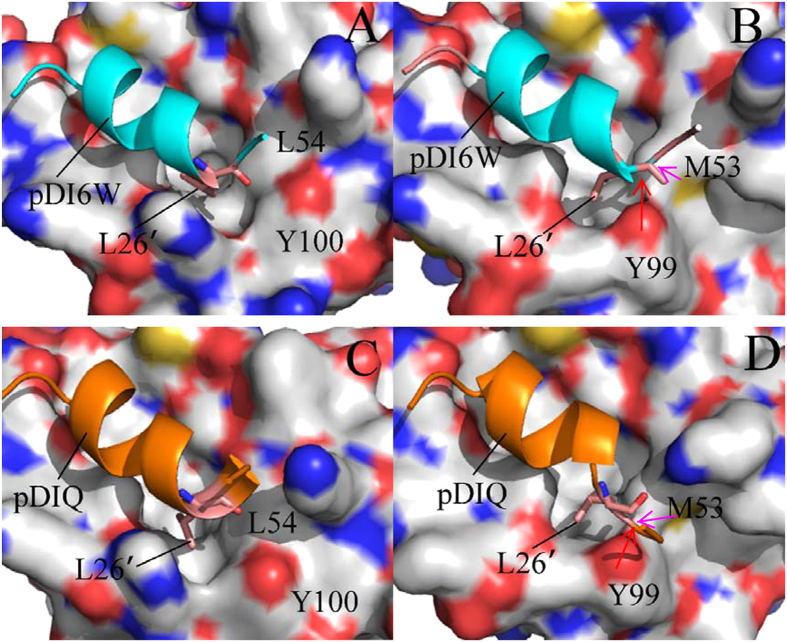
MDM2 and MDMX are displayed in surface modes, inhibitors in cartoon modes and Leu26′ in stick modes. (**A**) for the pDI6W-MDM2 complex, (**B**) for the pDI6W-MDMX complex, (**C**) for the pDIQ-MDM2 complex and (**D**) for the pDIQ-MDMX complex.

The above analyses suggest that the conformations of M53 and Y99 in MDMX are highly different from the one of L54 and Y99 in MDM2. The surface mode display of MDM2/MDMX (Fig. 9) structurally reveal that how these conformational difference affect the inhibitor bindings. By comparison of Fig. 9B,D with Fig. 9A,C, the long sidechain of M53 and the phenol of Y99 form two humps toward the residues L26′, which results in a potential steric clash with the residue L26′ and prevents L26′ from reaching the deep of the hydrophobic groove formed by the helix α2 and the helix α4 in MDMX (Fig. 1A). Thus, this potential steric clash reduces the number of van der Waals contacts of L26′ with the hydrophobic residues in the binding groove of MDMX.

**Figure 10:**
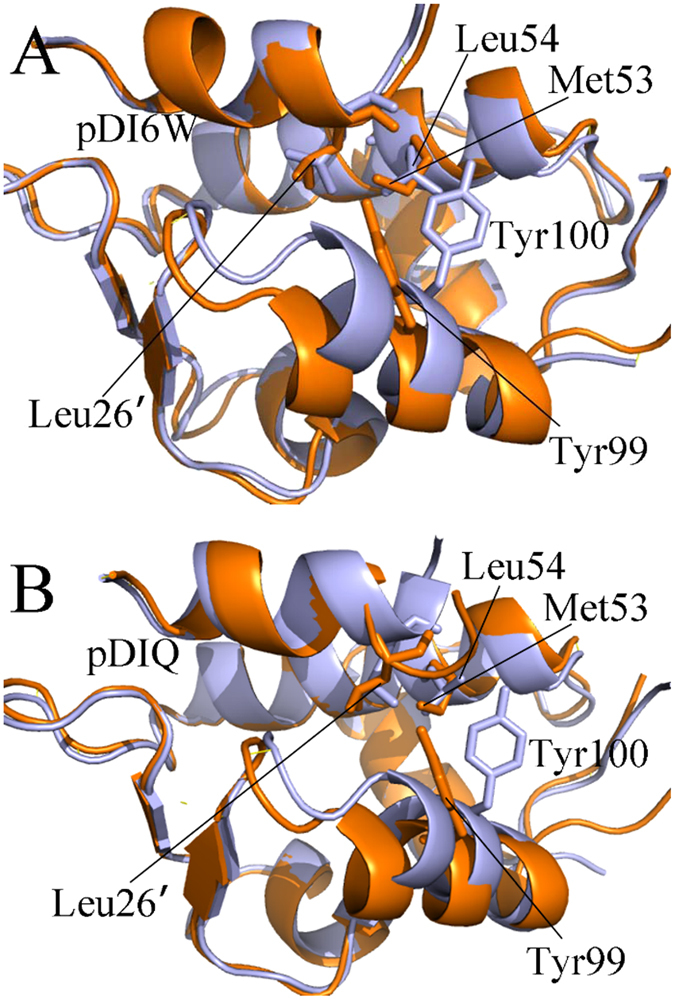
Superimposed structures of inhibitor-MDM2 complex and inhibitor-MDMX complex. MDM2/MDMX and inhibitors are shown in cartoon modes and the key residues in stick modes, MDM2 is displayed in light blue and MDMX in orange.

The above dihedral analyses suggest that the Chi1 angle of Tyr100 in MDM2 is about 186°, while the one of Y99 in MDMX is about 286°. The longer sidechain of M53 in MDMX relative to L54 in MDM2 points to the outside of the binding groove. To clearly clarify this difference, the structural superimposition analyses of MDM2 and MDMX were performed by using the lowest energy structure extracted from MD trajectory and depicted in Fig. 10. One can observe that the phenyl of Y100 flips away from the binding pocket and the residue L54 in the MDM2 (Fig. 10A,C). This orientation shows that the binding cleft of MDM2 shapes an open state, which allows L26′ and inhibitors to go into the deep of the cleft. Thus, L26′ can produce strong interactions with L54, V93, H96 and I99. However, the sidechains of M53 and Y99 in MDMX protrude into the binding groove, blocking a part of the cleft on its opposite sides and making the hydrophobic cleft smaller (Fig. 10B,D). This structural rearrangement of M53 and Y99 forms a closed conformation in MDMX, preventing the inhibitors and L26′ from reaching the deep of the binding cleft. Thus the van der Walls interactions of inhibitors with M53, V92, P95 and L98 in the hydrophobic groove of MDMX are reduced relative to that of inhibitors with the corresponding residues in MDM2, which agrees well with the analysis of the inhibitor-residue interactions. Overall, a conclusion can be obtained that the closed conformations of the binding cleft adopted by MDMX is the origin of weaker bindings of inhibitors to MDMX than to MDM2. Studies from Phan and Popowicz *et al*. also suggested that special conformation of M53 and Y99 leads to a steric clash with inhibitors, which agrees with our current results^19^,^77^.

## Discussions

In this work, the polarizable MD simulations of a combination FF02.r1 and POL3 and the non-polarizable MD simulations of the combination FF03 and TIP3P were performed to evaluate the effect of force field on the stability of protein second structure. The results indicate that the polarizable MD simulations can produce more hydrogen bonds and to stabilize the second structure. Meher and Zuo *et al*. also proved that the polarizable MD simulations can better maintain the hydrogen bonding interactions than the non-polarizable MD simulations^64^,^65^. Thus, the polarizable MD can provide rational conformations for the following post-processing analysis.

QM/MM-GBSA and SIE method were applied to calculate the binding affinities of inhibitors to MDM2 and MDMX. The predicted binding free energies using QM/MM-GBSA method quantitatively agree well with the experimentally determined values. The calculated results of two methods show that inhibitors produce weaker binding to MDMX than MDM2, and the decrease in the van der Walls interaction of inhibitors with MDMX relative to MDM2 is the main factor of weaker bindings of inhibitors to MDMX. Binding free energy predictions of Joseph *et al*. suggest that the reduction of van der Waals contacts induce weaker binding of inhibitors to MDMX^50^, which agrees with our current studies.

The time evolutions of dihedral angle Chi1 for M53 and Y99 in MDMX and L54 and Y100 in MDM2 were evaluated using polarizable MD trajectory. The results indicate that the conformations of M53 and Y199 are considerably different from L54 and Y100. The orientations of M53 and Y99 shape a closed conformation that prevents inhibitors from reaching the deep of the hydrophobic cleft in MDMX and reduce the van der Waals interactions of inhibitors with M53, V92, P95 and L98 in the cleft. Thus, the closed conformation of MDMX binding cleft caused by the orientations of M53 and Y99 is the main origin of weaker bindings of inhibitors to MDMX than to MDM2. In studies of Phan and Popowicz *et al*. the closed conformation constructed by the orientations of M53 and Y99 were also observed to affect the binding of inhibitors to MDMX^19^,^77^, which further supports our results. This study implies that it is essential how to relive the potential steric clash of inhibitors with the closed conformation of the binding cleft in MDMX for successful development of potent dual inhibitors targeting the p53-MDM2/MDMX interactions. Thus, during the development of inhibitors, the structure of inhibitors should be more flexible so that inhibitors can adjust its structure to meet the special conformation of MDMX and improve its binding abilities. We expect that the findings in our current studies can provide significant theoretical guidance for the designs of new-type dual inhibitors of anti-cancer treatment.

## Methods

### System setup

The crystal structures of MDM2/MDMX complexed with inhibitors pDI6W and pDIQ were obtained from the protein data bank (PDB): 3JZR for the pDI6W-MDM2 complex, 3JZP for the pDI6W-MDMX complex, 3JZS for the pDIQ-MDM2 complex and 3JZQ for the pDIQ-MDMX complex^19^. The protonation states of side chains in residues were determined at PH=7.0 by using PROPKA program^78^,^79^. The residues 26–108 in MDM2 and MDMX were used in studies and the terminal residues capped with an ACE and NME in the N- and C-terminus of proteins, respectively. All crystal water molecules were kept in the starting model. The leap module of Amber 12 program was used to add all missing hydrogen atoms to protein structures^80^. The force field parameters of MDM2/MDMX and inhibitors were assigned with ff03^81^ and ff02^82^ force fields to perform the non-polarizable and polarizable MD simulations, respectively. Cubic boxes of TIP3P^83^ water molecules were applied to solvate the protein complex in combination with the non-polarizable ff03 force field and that of POL3^84^ water molecules with the polarizable force field. The distance between the edge of the water box and the closest atom of solute was at least 12.0 Å. An appropriate number of counterions were added to neutralize the charge of each system.

### MD simulations

Initial bad steric contacts with the solvent or within protein molecules were removed by energy minimization of three steps. Firstly, 6000-step energy minimization, involving the steepest decent minimization of 3000 steps and conjugate gradient one of 3000 steps, was carried out in which a straining force was applied to all protein atoms. Then another 6000-step energy minimization was performed by refreezing all backbone atoms using the same restraining force. Finally, another 6000-step energy minimization was carried out without any restriction. Then, the system was heated from 0 to 300 K in 1 ns and equilibrated at 300 K for another 1 ns. Next, a 30-ns simulation without restriction was conducted at constant pressure and 300 K, and the conformations were saved every 2 ps. During MD simulations, the particle mesh Ewald (PME)^85^ method was employed to compute the long range electrostatic interactions using a 1.0 Å grid space and a fourth-order spline for interpolation. The non-bonded cutoff was set to 12.0 Å and the SHAKE method was used to restrain all bonds involving the hydrogen atoms^86^. The temperature was controlled using the Langevin dynamics with a collision frequency of 2 ps^−1^. Periodic boundary condition was adopted to avoid edge effect throughout MD simulations and a time step of 1 fs was set in all simulations.

### QM/MM GBSA calculations

The binding free energies of two inhibitors to MDM2/MDMX were calculated using QM/MM-GBSA approach in Amber based on 200 conformations extracted from the last 10 ns of MD trajectories with an interval of 50 ps^75^. The equations related with calculations of binding free energies was following.


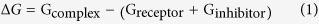



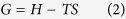



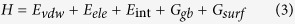


where Δ*G* is the total binding free energy, *G*_*complex*_, *G*_*receptor*_ and *G*_inhibitor_ are the free energies of the complex, receptor and inhibitor, respectively. *H* and *T*Δ*S* represent the enthalpy and entropy contributions of system. The terms *E*_*vdw*_, *E*_*ele*_ and *E*_*int*_ are van der Waals interaction, electrostatic interaction, and internal energy, respectively. The terms *G*_*gb*_ and *G*_*surf*_ are the polar and non-polar solvation free energy, respectively. *G*_*gb*_ is calculated using the modified GB model developed by Onufriev *et al*.^87^.*G*_*surf*_ is approximated by the experiential equation 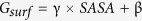, in which the values of *γ* and β were set to 0.0072 kcal. mol^−1^. Å^−2^ and 0.0 kcal · mol^−1^, respectively^88^. In this calculation, a probe radius of 1.4 Å for the solvent was adopted.

In the case of QM/MM-GBSA calculations, the enthalpy term was computed by applying the QM/MM approach. The residues involving the hydrogen binding interactions are described at the QM level using the semi-empirical Hamiltonian PM6 method to treat the hydrogen binding interactions. Our previous studies proved that this method can accurately evaluate the hydrogen bonding interactions^89^. All the rest of system were characterized at the MM level. The entropy changes upon inhibitor bindings were calculated by using a normal-mode analysis at the MM level^90^.

### Solvated interaction energy method

The SIE method was developed by Naim *et al*. to predict the binding affinities of inhibitors to proteins^71^. This method has been adopted to successfully study interaction modes of the other inhibitor-protein binding complexes and obtain rational results^91^,^92^. Thus, in this work, the SIE method was also used to calculate the binding free energies of inhibitors to MDM2/MDMX based on the following equation:


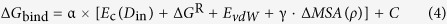


in which *E*_*c*_ and *E*_vdW_ are the intermolecular Coulomb and van der Waals interaction energies in the bound state, separately. These two terms were computed with the Amber ff02 force fields. The term ΔG^R^ represents the change of the reaction energy induced by inhibitor bindings and was calculated by solving the Poisson equation with the boundary element method (BRI BEM)^93^. The term γ. Δ*MSA* represents the change of the molecular surface area upon bindings. The Amber van der Waals radii linear scaling coefficient (ρ), the solute interior dielectric constant (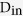), the molecular surface area coefficient (γ), the global proportionality coefficient related to the loss of conformational entropy upon binding (α) and a constant (*C*) are the parameters optimized by fitting to the absolute binding free energy for a set of 99 protein-ligand complex. The optimized values of these parameters are α = 0.1048, D_in_=2.25, γ = 0.0129 kcal/(mol · Å^2^) and *C* = −2.89 kcal · mol^−1^, respectively, which was used to successfully evaluate the binding modes of the other inhibitor-protein binding complexes^71^,^94^. The SIE calculations were performed with the program Sietraj^94^.

## Additional Information

**How to cite this article**: Chen, J. *et al*. Probing Origin of Binding Difference of inhibitors to MDM2 and MDMX by Polarizable Molecular Dynamics Simulation and QM/MM-GBSA Calculation. *Sci. Rep*. **5**, 17421; doi: 10.1038/srep17421 (2015).

## Supplementary Material

Supplementary Information

## Figures and Tables

**Table 1:** binding free energies calculated by QM/MM-GBSA method^a^.

Component	pDI6W-MDMX^c^	pDI6W-MDM2	pDIQ-MDMX	pDIQ-MDM2
ΔE_vdW_	−60.15 ± 0.40	−62.65 ± 0.38	−60.62 ± 0.52	−63.27 ± 0.36
ΔE_ele_	−141.91 ± 2.41	−242.39 ± 2.48	−145.64 ± 2.55	−287.07 ± 2.55
ΔG_gb_	190.73 ± 2.34	290.14 ± 2.35	195.45 ± 2.28	334.0 ± 2.58
ΔG_surf_	−7.95 ± 0.04	−8.37 ± 0.04	−8.01 ± 0.06	−8.84 ± 0.04
ΔG_qm_	−17.30 ± 0.39	−18.27 ± 0.36	−18.09 ± 0.49	−18.53 ± 0.39
ΔH_tot_	−36.58 ± 0.49	−41.54 ± 0.39	−36.91 ± 0.58	−43.71 ± 0.48
−TΔS	27.64 ± 0.48	30.52 ± 0.34	27.73 ± 0.62	31.89 ± 0.71
ΔG_bind_	−8.94	−11.02	−9.18	−11.82
^b^ΔG^exp^	−9.03	−10.50	−9.52	−11.11

^a^All energy values are given in kcal•mol^−1^.

^b^The experimental values of ΔG^exp^ were obtained by using the equation ΔG ≈ −RTlnIC50 based on the experimental IC50 values.

^c^Errors labeled by the signs  ±  represent the standard errors of mean.

**Table 2:** Binding free energies computed by the solvated interaction energy method^a^.

Component	pDI6W+MDMX	pDI6W+MDM2	pDIQ+MDMX	pDIQ+MDM2
ΔE_vdW_	−61.42 ± 0.20	−65.14 ± 0.17	−64.40 ± 0.21	−70.10 ± 0.17
ΔE_c_	−68.61 ± 0.80	−112.81 ± 0.85	−78.10 ± 0.55	−155.53 ± 0.65
γ·ΔMSA	−10.78 ± −0.03	−11.23 ± 0.02	−10.96 ± 0.02	−11.62 ± 0.02
ΔG^R^	70.23 ± 0.72	111.63 ± 0.78	81.18 ± 0.54	153.91 ± 0.59
C	−2.89 ± 0.00	−2.89 ± 0.00	−2.89 ± 0.00	−2.89 ± 0.00
ΔG_bind_	−10.28 ± 0.03	−11.01 ± 0.02	−10.46 ± 0.03	−11.60 ± 0.02
^b^ΔG^exp^	−9.03	−10.50	−9.52	−11.11

^a^All energy values are given in kcal•mol^−1^,

^b^The experimental values of ΔG^exp^ were obtained by using the equation ΔG ≈ −RTlnIC50 based on the experimental IC50 values.

**Table 3:** The hydrogen bonds formed between inhibitors and residues^a^.

complex	Donor	Acceptor	Distance(Å)	Angle(°)	Frequency(%)^b^
pDI6W-MDMX	F19′-N-H	Q71-NE1	2.92	135.12	53.34
	W23′-NE1-HE1	M53-OE1	2.88	1490.41	91.72
pDI6W-MDM2	F19′-N-H	Q72-NE1	3.01	138.45	71.64
	W23′-NE1-HE1	L54-OE1	2.87	150.36	99.09
pDIQ-MDMX	F19′-N-H	Q71-NE1	2.90	136.37	58.21
	W23′-NE1-HE1	M53-OE1	2.92	136.82	90.79
pDIQ-MDM2	F19′-N-H	Q72-NE1	2.89	139.51	60.06
	W23′-NE1-HE1	L54-OE1	2.89	148.18	98.96

^a^The hydrogen bonds determined by the acceptor…donor atom distance of less than 3.5 Å and acceptor…H-donor angle of greater than 120 Å,

^b^Occupancy (%) is given as the percentage of simulation time that a hydrogen bond exist.
