# Supplementary material for: Probing Origin of Binding Difference of inhibitors to MDM2 and MDMX by Polarizable Molecular Dynamics Simulation and QM/MM-GBSA Calculation
Source: Sci Rep. 2015 Nov 30;5:17421. doi: 10.1038/srep17421 (PMC4663504; doi:10.1038/srep17421)
Supplement: Supplementary Information [file srep17421-s1.pdf]

Probing Origin of Binding Difference of inhibitors to MDM2 and MDMX by Polarizable Molecular Dynamics Simulation and QM/MM-GBSA Calculation

Jianzhong Chen[a,*] ,Jinan Wang[b, *], Qinggang Zhang[c], Kaixian Chen[b], & Weiliang Zhu[b]

[a]School of Science, Shandong Jiaotong University, Jinan, 250014, China. Email:

[b]Discovery and Design Center, CAS Key Laboratory of Receptor Research, Shanghai Institute of Materia Medica, Chinese Academy of Sciences, 555 Zuchongzhi Road, Shanghai, 201203, China.

[c]College of Physics and Electronics, Shandong Normal University, Jinan, 250014, China.

[*]These authors contributed equally to this work.

Correspondence and requests for materials should be addressed to J.C. (chenjianzhong1970@163.com) and W.Z. (wlzhu@mail.shcnc.ac.cn.)

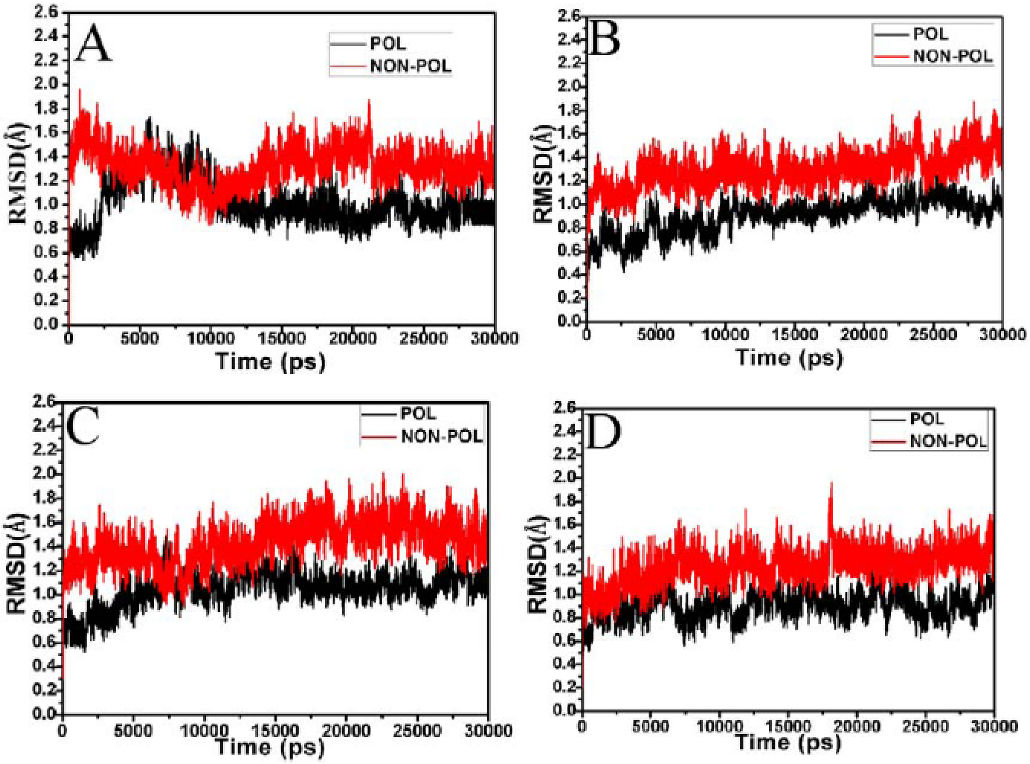

Figure S1 | Root –mean-square deviations (RMSD) of backbone atoms relative to their crystal structures. (A) for the pDI6W-MDMX complex, (B) for the pDIQ-MDMX complex, (C) for the pDI6W-MDM2 complex and (D) for the pDIQ-MDM2 complex. The non-polarizable ff03/TIP3P combination and the polarizable ff02.r1/POL3 combination are indicated by the black and red, respectively.

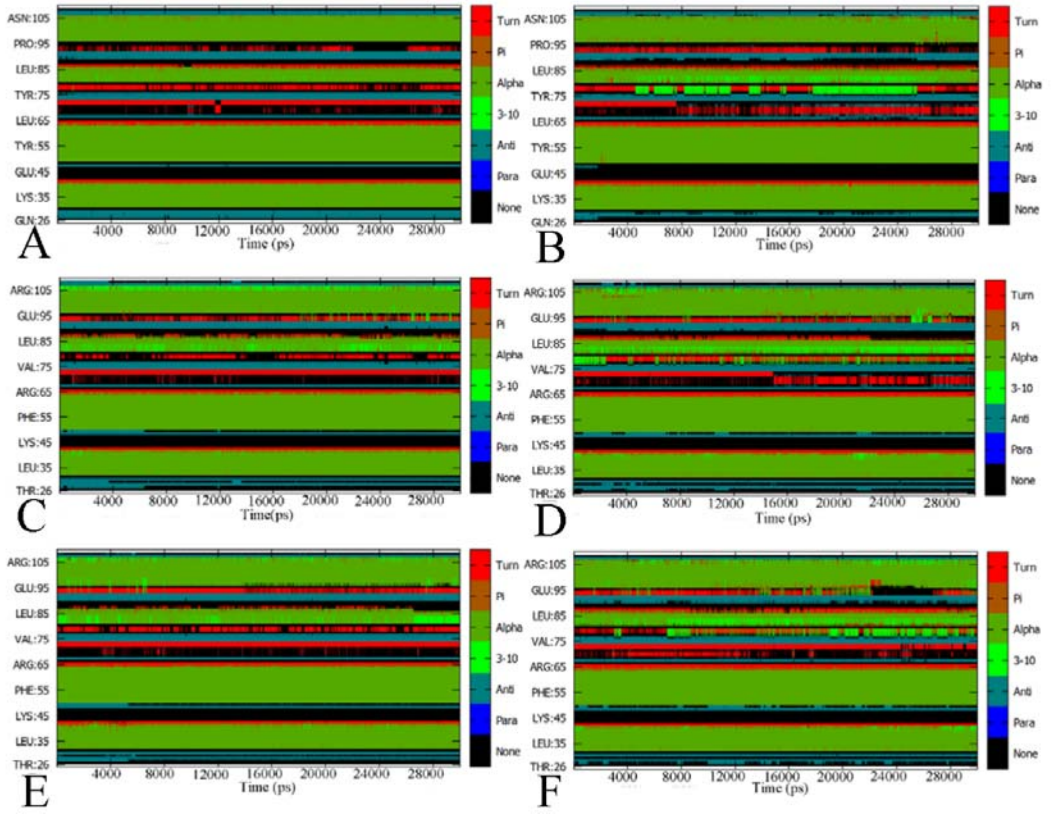

Figure S2 | Time evolution of the secondary structure profile of MDMX and MDM2: (A) for the polarizable ff02.r1/POL3 combination of the pDIQ-MDMX complex, (B) for the non-polarizable ff03/TIP3P combination of the pDIQ-MDMX complex; (C) for the polarizable ff02.r1/POL3 combination of the pD6W-MDM2 complex, (D) for the non-polarizable ff03/TIP3P combination of the pDI6W-MDM2 complex, (E) for the polarizable ff02.r1/POL3 combination of the pDIQ-MDM2 complex and (F) for the non-polarizable ff03/TIP3P combination of the pDIQ-MDM2 complex. Anti and Para represent anti-parallel beta-sheet and parallel beta-sheet, respectively, while Pi, 3-10 and Alpha represent Pi (3-14) helix, 3-10 helix and alpha helix.

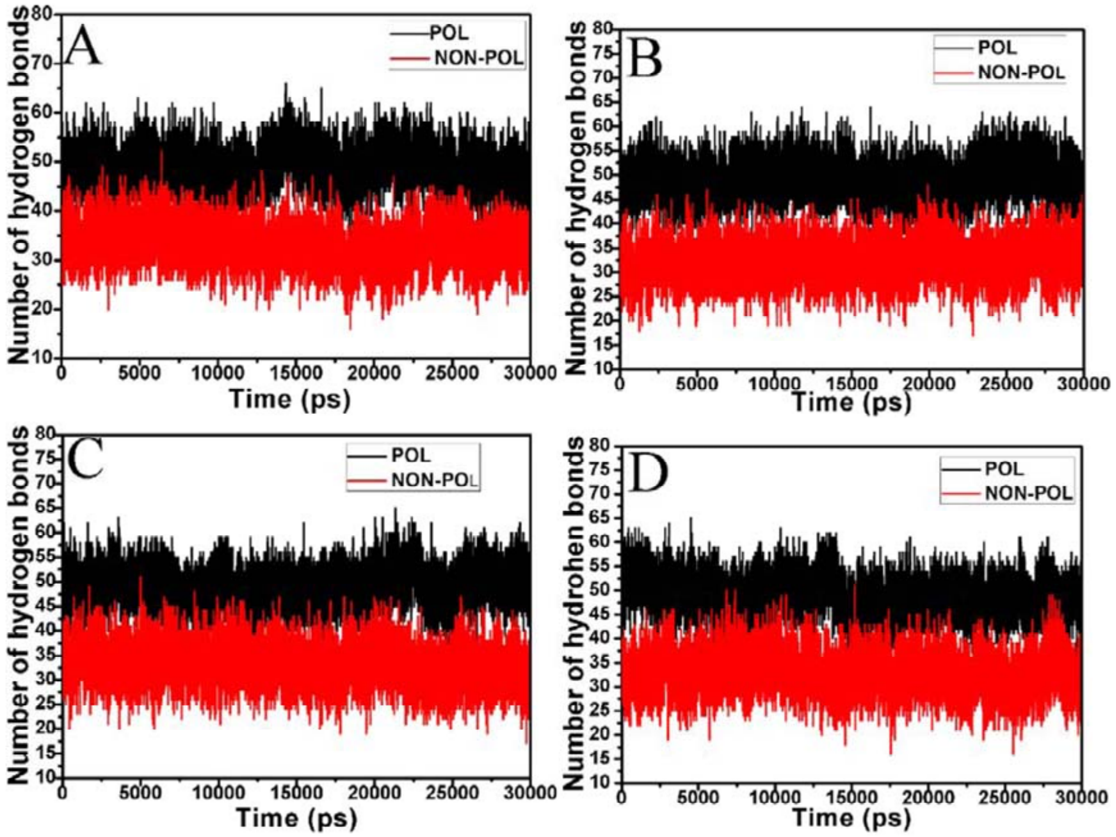

Figure S3 | Time evolution of hydrogen bond numbers in the binding complexes: (A) for the pDI6W-MDMX complex, (B) for the pDIQ-MDMX complex, (C) for the pDI6W-MDM2 complex and (D) for the pDIQ-MDM2 complex. The polarizable ff02.r1/POL3 combination and the non-polarizable ff03/TIP3P combination are labeled by the black and red, respectively.

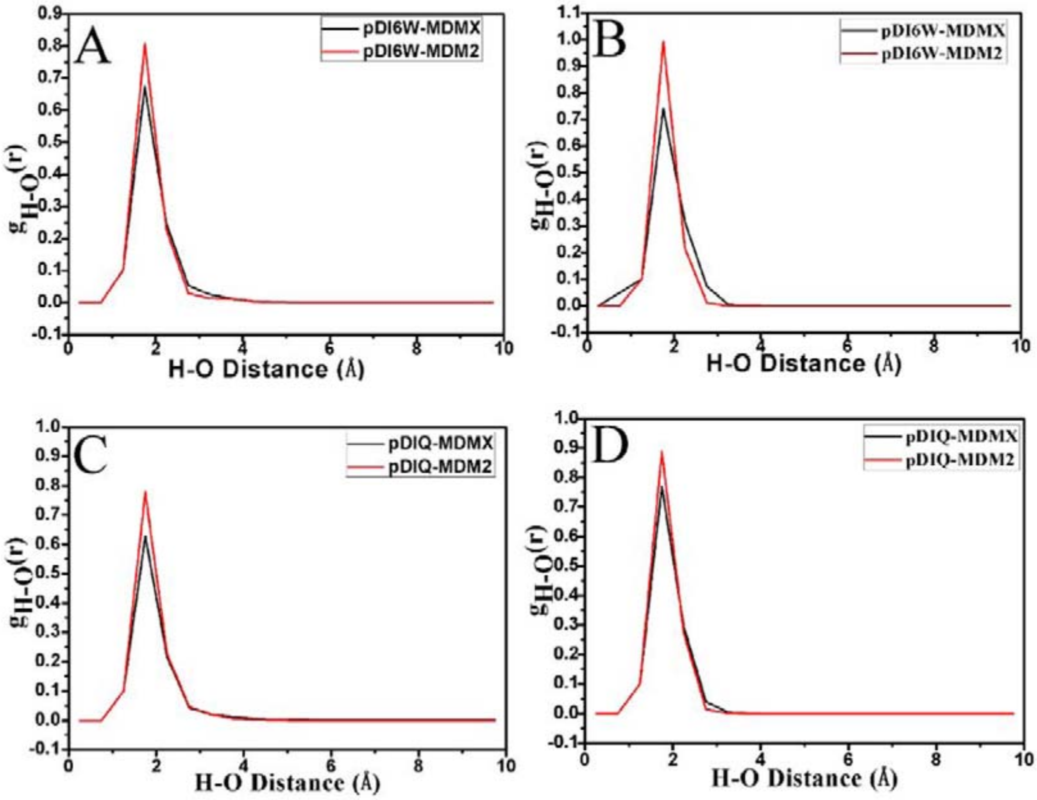

Figure S4 | Radial distribution function of H–O distance for hydrogen bonds: (A) for the hydrogen bond M53O…W23´NE1-HE1(pDI6W), (B) for the hydrogen bond Q72OE1…F19´N-H(pDI6W), (C) for the hydrogen bond M53O…W23´NE1-HE1(pDIQ) and (D) for the hydrogen bond Q72OE1…F19´N-H(pDI6W).

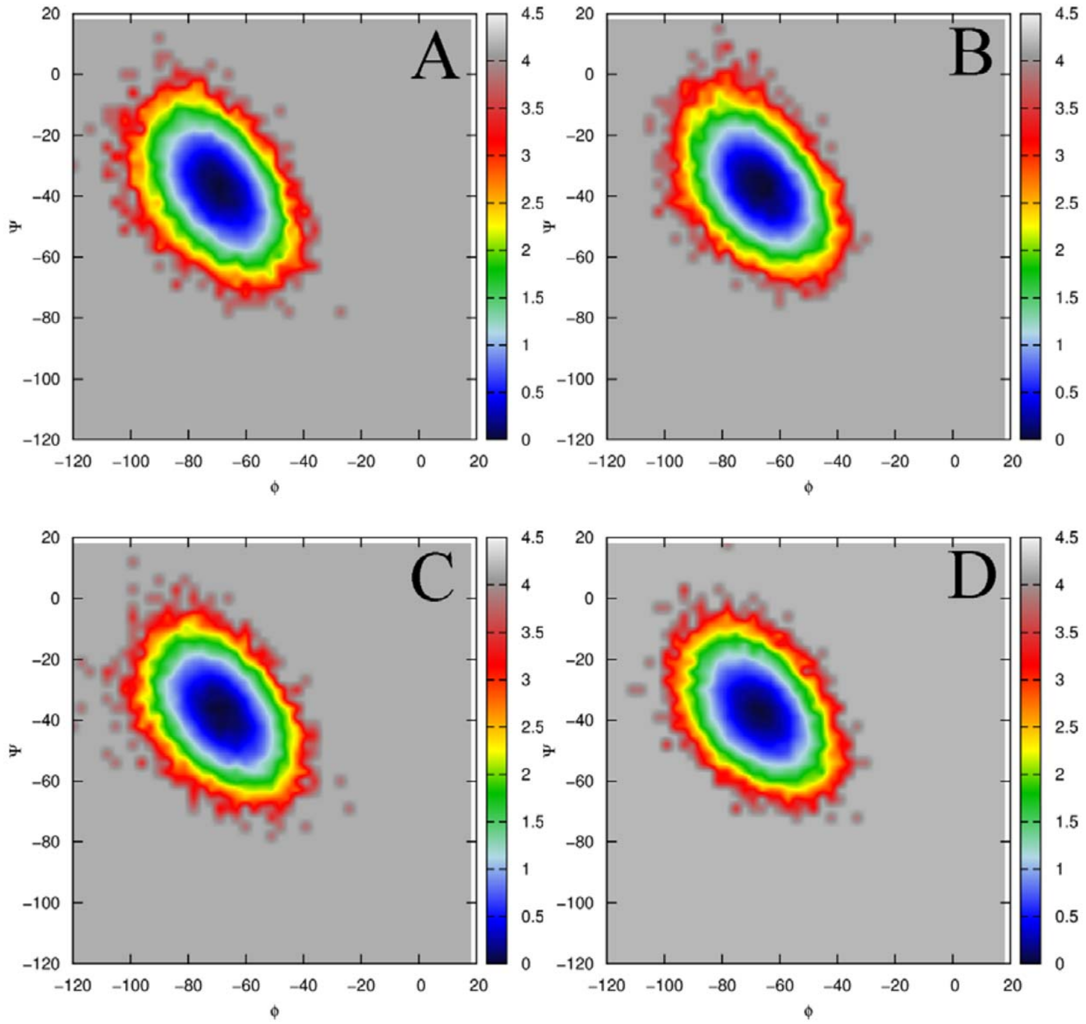

Figure S5 | Free energy contour map as function of the backbone angle ψ and φ. (A) for L54 in the pDI6W-MDM2 complex, (B) for M53 in the pDI6W-MDMX complex. (C) for L54 in the pDIQ-MDM2 complex and (D) for M53 in the pDIQ-MDMX complex.
